# Involvement of 5-lipoxygenase/cysteinyl leukotriene receptor 1 in rotenone- and MPP+-induced BV2 microglial activation

**DOI:** 10.1186/1750-1326-7-S1-S24

**Published:** 2012-02-07

**Authors:** Yanfang Wang, Xiaoyan Zhang, Chengtan Li, Jianbo Zhao, Erqing Wei, Lihui Zhang

**Affiliations:** 1 Hangzhou Key Laboratory of Neurobiology and Department of Pharmacology, School of Basic Medicine, Hangzhou Normal University, Hangzhou 310036, China; 2 Department of Pharmacology, School of Medicine, Zhejiang University, Hangzhou 310058, China

## Background

Neuroinflammation plays a prominent role in the pathogenesis of Parkinson’s disease (PD), and microglial activation contributes to initiating and maintaining brain inflammation and neuronal death. 5-Lipoxygenase (5-LOX) is a key enzyme catalyzing arachidonic acid to produce cysteinyl leukotrienes (CysLTs). CysLTs are potent proinflammatory mediators, and their actions are mediated by activating CysLT receptors. We recently reported that rotenone time- and concentration-dependently induced 5-LOX translocation into the nuclear envelope (a key event for 5-LOX activation) and cell injury in PC12 cells, and the 5-LOX selective inhibitor zileuton attenuated rotenone-induced 5-LOX activation and cell injury. To determine the role of 5-LOX pathway in microglial-dependent neuroinflammation, we investigated the changes of 5-LOX and CysLT_1_ receptor in a cell model of PD induced by specific mitochondrial complex I inhibitors (rotenone or 1-methyl-4-phenylpyridinium (MPP^+^)) in BV2 microglial cells.

## Methods

BV2 cells, a murine BV2 microglia cell line, were cultured in media with or without rotenone (0.1, 0.3, 1, 3, 10 nM) or MPP^+^ (0.003, 0.01, 0.03, 0.1, 0.3 μM) for 24 h. The number of microglia was counted. Phagocytotic activity of BV2 cells was evaluated using fluorescent microspheres. Expression and translocation of 5-LOX and CysLT_1_ receptor were detected by immunocytochemical analysis.

## Results

The low doses of rotenone (1-10 nM) or MPP^+^ (0.03-0.3 μM) induced cell proliferation and microglial phagocytosis in BV2 cells. The number of BV2 cells was significantly increased after 24 h treatment with 1 nM rotenone or 0.03-0.1 μM MPP^+^. After treatment with 1-10 nM rotenone or 0.01-0.3 μM MPP^+^, phagocytosis was significantly increased in BV2 cells. Furthermore, we found that 5-LOX expression was increased in a time-dependent manner, and 5-LOX was primarily localized in the nuclear envelope and cytoplasm, and a plaque-like distribution was found in the nucleus in rotenone (3 nM)-activated BV2 cells. In addition, MPP^+^ (0.003-0.3 μM) concentration-dependently induced CysLT_1_ receptor translocation from cell membrane to the cytoplasm.

## Conclusion

These results suggest an involvement of the 5-LOX/CysLT_1_ receptor in rotenone- and MPP^+^-induced BV2 microglial activation. The 5-LOX signaling pathway might therefore be a potential therapeutic target for modulating microglial-mediated inflammation of PD.

